# The Evolution of Spinal Endoscopy: Design and Image Analysis of a Single-Use Digital Endoscope Versus Traditional Optic Endoscope

**DOI:** 10.3390/bioengineering11010099

**Published:** 2024-01-20

**Authors:** Shih-Hao Cheng, Yen-Tsung Lin, Hsin-Tzu Lu, Yu-Chuan Tsuei, William Chu, Woei-Chyn Chu

**Affiliations:** 1 Institute of Biomedical Engineering, National Yang-Ming Chiao-Tung University, Taipei 11221, Taiwan; franchpaladin.y@nycu.edu.tw (S.-H.C.); jason@chiyitech.com (Y.-T.L.); dontspider@yahoo.com.tw (Y.-C.T.); 2 Department of Orthopedics, Cheng Hsin General Hospital, Taipei 11221, Taiwan; 3 School of Nursing, National Taipei University of Nursing and Health Sciences, Taipei 11221, Taiwan

**Keywords:** image comparison, minimally invasive surgery, single-use digital endoscope, spinal endoscopy, surgical site infection prevention

## Abstract

Spinal endoscopy has evolved significantly since its inception, offering minimally invasive solutions for various spinal pathologies. This study introduces a promising innovation in spinal endoscopy—a single-use digital endoscope designed to overcome the drawbacks of traditional optic endoscopes. Traditional endoscopes, despite their utility, present challenges such as fragility, complex disinfection processes, weight issues, and susceptibility to mechanical malfunctions. The digital endoscope, with its disposable nature, lighter weight, and improved image quality, aims to enhance surgical procedures and patient safety. The digital endoscope system comprises a 30-degree 1000 × 1000 pixel resolution camera sensor with a 4.3 mm working channel, and LED light sources replacing optical fibers. The all-in-one touch screen tablet serves as the host computer, providing portability and simplified operation. Image comparisons between the digital and optic endoscopes revealed advantages in the form of increased field of view, lesser distortion, greater close-range resolution, and enhanced luminance. The single-use digital endoscope demonstrates great potential for revolutionizing spine endoscopic surgeries, offering convenience, safety, and superior imaging capabilities compared to traditional optic endoscopes.

## 1. Introduction

The introduction of endoscopy in spinal procedures dates back to the 1970s, pioneered by Kambin and Hijikata [1]. Dr. Yeung later introduced Yeung endoscopic surgery in the 1990s, marking a significant milestone in spinal endoscopic advancements, also known as the Yeung endoscopic spine system (YESS) [2]. Initially, the spinal endoscope focused on addressing intervertebral disc disorders like protrusions and ruptures of the intervertebral disc [3]. Herniated lumbar discs may cause severe axial back pain and radicular pain, which may require surgical removal. Percutaneous endoscopic lumbar discectomy (PELD) emerged as a procedure to alleviate symptoms by removing the compressing intervertebral disc. Traditionally, discectomy was performed through open surgery under general anesthesia and assisted by a microscope or loupe. The invention of the uni-portal spinal endoscope allowed for minimally invasive procedures under local anesthesia, involving only a small stab wound, leading to reduced post-operative pain and faster recovery [4]. Subsequent innovations, such as the unilateral biportal endoscope (UBE) technique, interlaminar and paraspinal approach for full endoscopic surgery, introduced new indications. Unlike the uni-portal endoscope, UBE allows instruments to enter the body from a separate wound, offering enhanced flexibility [5]. Specific endoscopes of varying diameters and lengths were created for these different surgical methods. These different surgical techniques, along with advanced endoscopic instruments like burrs and rongeurs, have broadened the applications of spinal endoscopic surgery to encompass diverse spinal conditions, including infections, degenerative spondylosis, and spinal fusion surgery [6]. The discussion of the spine endoscope and the introduction of new techniques has surged in popularity in recent studies [7,8,9]. Numerous research findings suggest that, in comparison to traditional open surgery, spinal endoscopic surgery not only minimizes soft tissue damage but also achieves comparable outcomes [10,11,12].

Traditionally, although the design of endoscopes varies among manufacturers, there are some common fundamental elements. An optical endoscope comprises a metal shell and lenses with different angles. The endoscope’s angle of lens is specifically crafted to accommodate the narrow surgical space and limited approach angles. Surgeons can enhance their field of view and observe structures not easily visible by rotating the endoscope. The proximal observation window on the endoscope is typically linked to a camera head, converting analog signals to digital signals sent to an endoscope console. The resulting images and videos are displayed on a high-resolution medical monitor, guiding the surgeon during the procedure. An additional light source, such as Xenon or Halogen light, transmits strong light to the endoscope’s tip through an optical fiber. In spinal endoscopic surgery, irrigation fluid, commonly aseptic saline or distilled water, is essential for maintaining the surgical space, removing tissue debris, and controlling bleeding. The fluid pressure aids in reducing active bleeding and ensuring clear visibility. These components, including the endoscope, endoscope console, light source, monitor, irrigation fluid, and control computer, collectively form a standard endoscopic system [13,14]. The system is delicate, requiring careful protection, storage, and the expertise of professional staff for preparation and setup before surgery.

Despite the advancements in spine endoscopy, traditional optic endoscopes still present numerous inherent drawbacks. The high cost associated with optical endoscopes has led to their repetitive use becoming a standard practice. Most endoscopes are delicate and require a low-temperature disinfection method, with liquid chemical disinfection being the most commonly used and cost-effective approach [15,16]. This process is time consuming, and concerns exist about the incomplete elimination of fungal spores and biofilms [17,18,19]. According to the past literature, the chances of infection following spinal endoscopy range from 0.001% to 1.4% [20,21,22]. Bacterial infections constitute the majority of these cases, and the most common pathogenic bacteria have not been conclusively determined. Infections caused by *Pseudomonas*, *E. coli*, and *Staphylococcus* have all been reported [23]. Outbreaks of nosocomial infections associated with endoscopes have been periodically reported, despite the existence of guidelines established by various federal agencies for endoscope reprocessing [24,25]. The primary reasons for these infection outbreaks are often attributed to the repeated use of endoscopes and failures in manual disinfection. The intricate design and the presence of narrow and elongated channels in the endoscope itself further complicate thorough disinfection, potentially leading to the accumulation of patient tissues on the luminal walls, resulting in contamination and toxic reactions [26,27]. Additionally, issues such as malfunctions in automated endoscope processors and equipment defects contribute to these events [24]. Furthermore, spine surgery poses distinctive infection risks due to specific factors. Poor blood supply in the intervertebral disc limits the body’s natural defense [28]. Osteomyelitis, a bone infection common in spine surgery, is resistant to antibiotics, complicating treatment. Implantation in spine surgeries introduces foreign materials, increasing infection risks as bacteria may form biofilms on implants. These factors elevate infection risks in spine surgery compared to other procedures. Surgical site infection not only poses a threat to patient safety but also results in increased healthcare costs and the heightened spread of drug-resistant pathogens.

There are other disadvantages of tradition endoscopes in addition to disinfection concerns. Traditional endoscopes consist of a metal casing, prism, and lens, contributing to a significant overall weight for the device. Additionally, there are extra wires, optical fibers, and a charge-coupled device (CCD) connected to the endoscope. Moreover, the current design does not align with ergonomics, leading to increased fatigue and occupational hazards for surgeons with prolonged usage [29]. The size of the optic endoscope system is substantial, necessitating a cart to store all the equipment. Transporting and setting up the entire system require considerable effort. The limited field of view in endoscopes necessitates an increase in the forward lens angle and repeated rotation of the endoscope to enlarge the field of view. Lastly, lens scratching, fogging, and mechanical malfunctions occur during operations from time to time [30]. Considering the high device cost, many hospitals do not have many reserved devices. When faced with such conditions and when immediate problem resolution is not possible, patient safety may be compromised.

Given the mentioned drawbacks, our objective was to develop a lightweight, single-use spine endoscope with improved imaging capabilities. A video sensor was integrated into a disposable rigid spine endoscope. Utilizing a high-resolution sensor with a wide viewing angle in a disposable rigid spine endoscope with a working channel is a novel approach that has not been previously documented. No study to date has directly compared its efficacy and image quality to that of an optic endoscope. Our research involved a comprehensive comparison of various parameters between two types of spine endoscopes. We believe that such a design could enhance the efficiency of spine endoscopic surgeries and elevate patient safety.

## 2. Materials and Methods

### 2.1. Design of the Endoscope System

**Figure 1:**
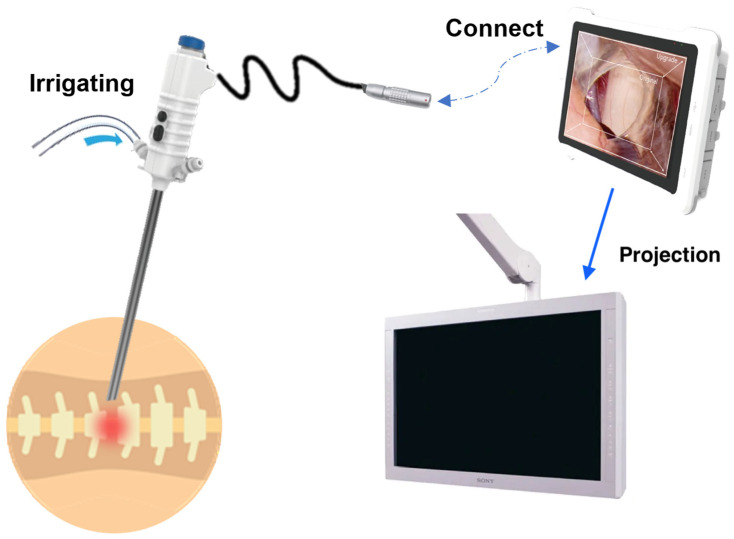
A depiction of the digital spine endoscope system: a single-use digital endoscope connected to a medical tablet with a touch screen. The option to project the image onto a larger medical monitor is also available. The built-in channel is connected to an irrigation fluid and suction tube.

The diagram below (Figure 1) illustrates the whole system. We have substituted the optical endoscope system with a digital disposable endoscope. Illumination for the camera is provided by a light-emitting diode (LED) located at the endoscope’s front, eliminating the necessity for an external light source and optical fibers. A single integrated disposable wire is responsible for both electrical and image transmission functions, showcasing a notable improvement in flexibility and weight compared to traditional wires and optic fibers. The endoscope console with a touch screen is also more lightweight and portable, enhancing both input and output capabilities. Furthermore, the tablet has the capacity to relay its video output to a larger, high-resolution medical monitor. To sum up, this entire system offers increased convenience and reduced weight in comparison to the existing system.

### 2.2. Design of Endoscope Body and Distal Camera

**Figure 2:**
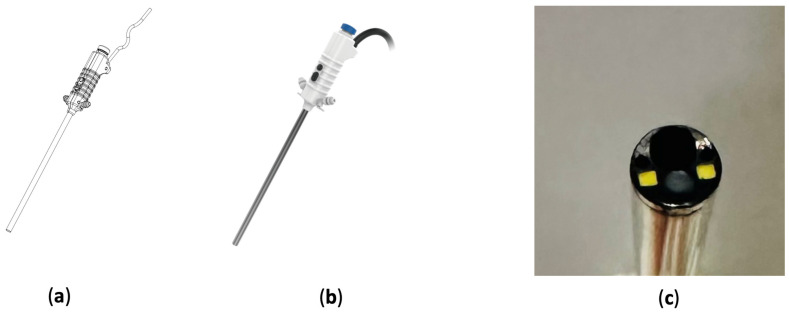
Illustration of the digital spine endoscope: (**a**) CAD image, (**b**) appearance, and (**c**) tip of the endoscope. The front of the endoscope features a camera, two LED lights, two irrigation channels, and a working channel.

We employed a conventional 30-degree uni-portal optic endoscope (ShenDa^®^, Shenyang, China) as a reference to establish the specifications and camera angle for our digital endoscope. As a result, we developed a 30-degree endoscope with an integrated 4.3 mm working channel, using computer-aided design (CAD) (Figure 2a). Instead of using conventional optical fiber, we incorporated dual LED lights at the endoscope tips to serve as the light source. The LEDs have a nominal correlated color temperature of 3500 K and a brightness of 64 Lumens. The forward voltage is 3.3 V and the forward current is 10 μA. The viewing angle of the LEDs is 170 degrees. The endoscope is equipped with a Complementary Metal-Oxide-Semiconductor (CMOS) image sensor at the front, featuring a pixel count of 1000 × 1000 pixels and pixel dimensions of 1.4 μm × 1.4 μm. The sensor operates at a frame rate of 80 frames per second (FPS). The plastic part of the endoscope’s body was fabricated using polycarbonate through 3D printing photopolymerization, while the metallic parts are made from stainless 316, providing good corrosion resistant and mechanical strength. To facilitate irrigation, there are two channels on both sides of the endoscope tip. The top of the endoscope handle includes buttons for white balance adjustment, screen capture, and video recording. A single electrical wire is positioned at the endoscope’s base, serving both power supply and signal transmission. The disposable wire, with a diameter of 4.5 mm and a length of 2 m, underwent rigorous testing for open, short, and missing wires. The conductor resistance is a maximum of 3 Ω/km and the insulation resistance is a minimum of 10 MΩ/km. The wire has a dielectric withstand voltage of 100 v dc/10 ms. All specifications comply with RoHS environmental standards. The wire is aseptic and disposable, eliminating the need for an additional plastic endoscope cover. The endoscope also supports plug-and-play functionality, allowing for easy replacement during surgery. A visual representation and cross-sectional view of the endoscope are presented in the accompanying illustration (Figure 2).

### 2.3. Monitor and Endoscope Console

This tablet features a 10-inch touchscreen with a resolution of 1600 × 2170 and weighs 1510 g. It can be powered by a 12 V, 2 A alternating current and is equipped with a built-in 15 V 6500 mAh lithium-ion battery, providing a runtime of 4 h. It adopts standard Lemo connectors for stable and reliable connection. In addition, it has an HDMI output port that can project the image onto a medical screen for convenient observation by doctors and other medical staff. There is also a USB port for image transfer, which can be used to save the images and videos of the endoscopic examination to a computer or other device. This product, moreover, is customized to meet specific needs. This compact computer is both portable and lightweight compared to traditional systems, which involve separate computers and endoscope consoles. The touch screen eliminates the need for a mouse and keyboard, streamlining the system even further. The tablet is compatible with medical screens, allowing high-resolution video projection. The integrated software supports video recording and photo capture, improving the overall user experience. Users can make real-time adjustments to image settings, such as brightness, contrast, and color, all on the same platform. Additionally, the patient’s data can be directly saved to storage devices or uploaded to the medical image viewer within a medical facility. This comprehensive design aims to boost efficiency and convenience in medical imaging procedures.

### 2.4. Image Captures

**Figure 3:**
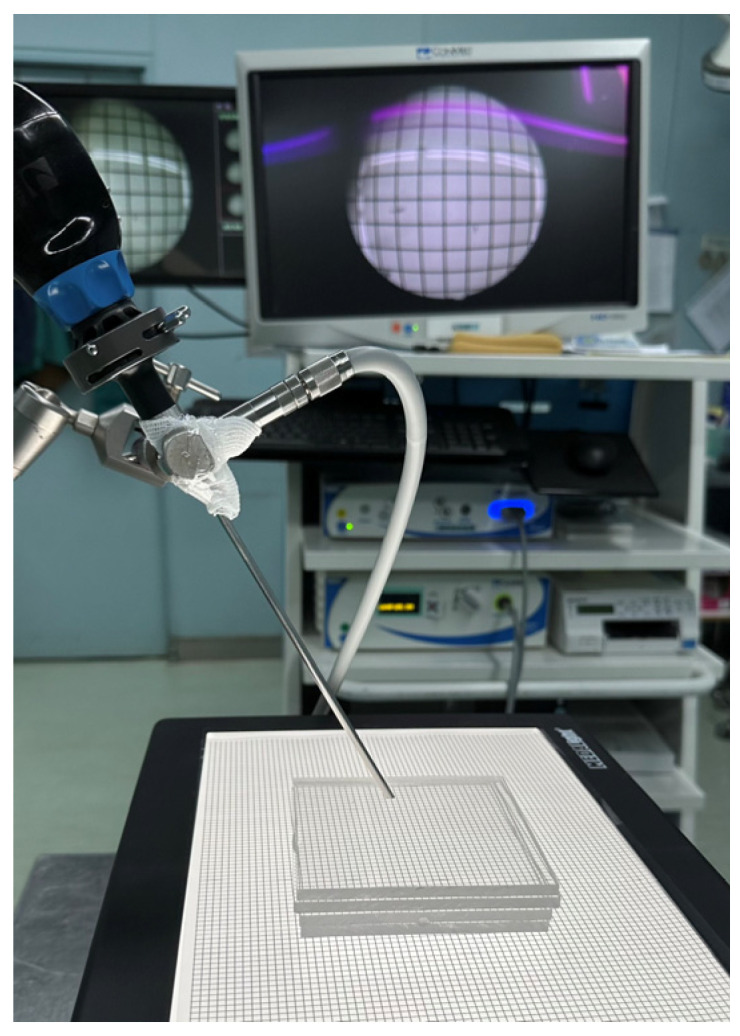
The setup for chart photography. The endoscope was secured on a holder, with the distal lens perpendicular to the ground. Plastic blocks were used to confirm the distance between the tip and the chart. After removing the block, the lights were turned off and the image was captured for analysis.

We conducted a comprehensive image analysis for both the digital and reference optical endoscopes. For a controlled experiment setup, we built a darkroom with backlighting and conducted a series of experiments employing diverse charts tailored to specific testing objectives. The endoscope was securely positioned on a holder in the darkroom, ensuring that the visual field was perpendicular to the ground. Different standard charts were illuminated by an LED backlight, and images were captured at normal magnification, without zooming in or out using both the optical and digital endoscopes. The distance between the tip of the endoscope and the chart was confirmed using plastic blocks, each 5 mm thick, and then reconfirmed with a ruler after the blocks were removed (Figure 3).

The test was calibrated using white balance before it began. In the visual field test and grid test, the endoscope was placed inside a multi-color temperature lightbox, positioned at 30 mm with a 6500 K LED light source, and images were captured. Grid ground paper was utilized to calculate the visual field and distortion, with the analysis conducted in both room air and underwater to realistically replicate surgical conditions. Photographs were taken at varying distances to assess image performance under different conditions. In the USAF 1951 resolution test, the endoscope was positioned at 3 mm/5 mm/10 mm/15 mm/20 mm positions and images were captured. For all other electronic image files, MTF tests were performed using the SFRplus Chart (3nh^®^, Shenzhen City, China) positioned at 10 mm/50 mm/100 mm. Grayscale was evaluated using the TE241 OECF20 (3nh^®^, Shenzhen City, China) and Noise Chart (3nh^®^, Shenzhen City, China), displayed full-screen on the monitor. Color difference was evaluated using the TE188 Color Rendition Chart (3nh^®^, Shenzhen City, China), also displayed in full screen. Brightness uniformity was evaluated using a gray test card placed at a distance of 20 mm. The image tests with backlight were processed and analyzed using Imatest software 3.7 (Imatest^®^, Boulder, CO, USA).

### 2.5. Images Analysis and Comparison with a Traditional Optic Endoscope

At various distances and environmental conditions, both endoscopes captured 30 images of a single chart. In the visual field test and grid test, two investigators independently counted the results and calculated the average. In the USAF 1951 resolution test, two investigators individually examined the photos; in cases of discrepancy, a third investigator was consulted to establish a consensus. All other electronic image files underwent processing and analysis using Imatest software 3.7 (Imatest^®^, Boulder, CO, USA). Imatest, a commercial software widely recognized for its effectiveness, has been applied in many prior studies focusing on image processing [31,32,33]. A comprehensive comparative analysis was conducted, evaluating the image distortion, resolution, color saturation, luminance, and grayscale performance of the two devices.

### 2.6. Software Analysis Algorithms

#### 2.6.1. Distortion Correction

The SMIA TV distortion value quantifies the perceived warping of an image as a percentage. This measurement is based on the difference between the heights of the image corners and the center, reflecting how much the image deviates from a perfectly flat, undistorted representation. We used 3rd order distortion equation correction coefficients to calculate distortion rates. The software calculated the distortion ratio using the following formula:

(1) $$r_{u}=r_{d}+k_{1}r_{d}^{3}$$

where $r_{u}$ and $r_{d}$ represent the undistorted and distorted radii, respectively.

#### 2.6.2. Modulation Transfer Function

The Modulation Transfer Function (MTF) is a crucial measure of image sharpness, typically presented as a function of spatial frequency. It assesses the imaging system’s capacity to reproduce contrast at higher spatial frequencies. The modulation of the captured image can be determined by calculating the ratio of the fundamental frequency component to the DC (Direct Current) component, expressed as:

Modulation (f) = Fundamental frequency component/DC component.(2)

The resolution conversion method for the image sensor was determined using the following formula:

(3) $$N_{f}=\frac{picture\;height\;(mm)}{pixel\;size}$$

$N_{f}$ denotes the resolution limit and adheres to the Nyquist sampling theorem.

#### 2.6.3. Color Difference

Color difference examines images with the commonly used 24-patch GretagMacbeth ColorChecker (3nh^®^, Shenzhen City, China). The last row (patches 19–24) contains six grayscale patches ranging from white to black, with densities from 0.05 to 1.5. The CIEDE2000 formulas, widely acknowledged as the superior color difference metric, surpass their predecessors in accuracy. Color differences are expressed in the CIELAB color space, where L* denotes luminance, a* represents color on a green–red scale, and b* signifies color on a blue–yellow scale. The Imatest software calculated Delta *E* ($∆E_{ab}^{*}$) using the following equation:

(4) $$∆E_{ab}^{*}=\sqrt{{{(L}_{2}^{*}-L_{1}^{*})}^{2}+{{(a}_{2}^{*}-a_{1}^{*})}^{2}+{{(b}_{2}^{*}-b_{1}^{*})}^{2}}$$

Delta *C* ($∆C^{*}$) was calculated using the following equation and follows a similar pattern:

(5) $$∆C^{*}=\sqrt{{{(a}_{2}^{*}-a_{1}^{*})}^{2}+{{(b}_{2}^{*}-b_{1}^{*})}^{2}}$$

Nevertheless, in contrast to $∆E_{ab}^{*}$, it excludes the consideration of luminance difference in the computation.

#### 2.6.4. Luminance Uniformity

This assessment involves creating normalized pixel-level contours for the luminance channel of the image. Luminance, calculated as 0.2125R + 0.7154G + 0.0721 × B, where R, G, and B represent the red, green, and blue color channels, assigns a maximum value of 1. This corresponds to a pixel level of 255 for image files with an 8-bit depth or 65,535 for those with a 16-bit depth.

#### 2.6.5. Gray Scale

The Imatest image analysis software’s Stepchart module was employed for processing images captured from the 20-patch OECF targets chart. The camera distance to the chart was adjusted to ensure an approximate horizontal resolution of 50 pixels per patch, as specified by the Stepchart requirements.

### 2.7. Statistics

The data underwent processing using SPSS 21 (IBM, New York, NY, USA). For the measurement of SMIA TV distortion, SFR, color difference, and luminance, the data were input into software for analysis. The average value was computed and subjected to an independent Student’s T test between two groups, with a *p*-value of <0.05 considered statistically significant.

## 3. Results

### 3.1. Product Dimensions and Specifications

The digital endoscope shares identical dimensions with traditional endoscopes, measuring 181 mm in length. It features an outer diameter of 8.5 mm, slightly larger than that of optical endoscopes. The working channel diameter remains consistent, enabling the utilization of existing surgical instruments and eliminating the need for a new set of tools. The weight of the digital endoscope, excluding additional wires, is 39 g, while the traditional endoscope weighs 214 g. In a standard surgical setup, the measured weight was 43 g for the digital endoscope and 439 g for the optical endoscope. The notable difference in weight primarily arises from the camera head and the optical fiber.

### 3.2. Field of View

**Figure 4:**
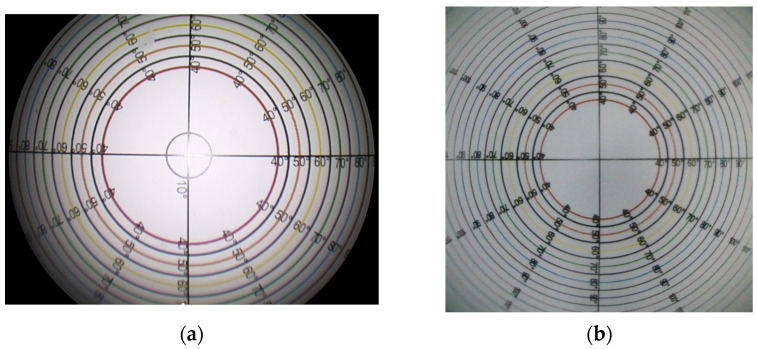
Images captured during the viewing angle experiment using (**a**) the optical endoscope and (**b**) the digital endoscope. The field of view for an optical endoscope is circular, while the field of view for an digital endoscope is square. Each concentric circle of a different color represents a different viewing angle. The optical endoscope had a fixed angle of 89 degrees, whereas the digital endoscope provided a variable range from a minimum of 100 degrees to a maximum of 130 degrees.

The fundamental difference lies in the shape of the visual field. The traditional endoscope provides a circular visual field with a similar viewing angle from each direction. In contrast, the digital endoscope offers a rectangular or square visual field, characterized by three measurements: horizontal, vertical, and diagonal view angles (Figure 4). The viewing angle for the optical endoscope was 89.2 ± 1.23 degrees. In the case of the digital endoscope, the angle was 100.1 ± 1.32 degrees for both width and length, and 130.6 ± 1.87 degrees in the diagonal direction. The overall viewing angle surpassed that of the traditional endoscope (*p* < 0.001).

### 3.3. Grid Ground Test

The grid ground tests were conducted at distances of 10 mm, 20 mm, and 30 mm between the endoscope tip and the subjects in ambient air. These distances are commonly encountered in surgical procedures, with each grid representing a size of 5 mm × 5 mm. The digital endoscope displayed a significant greater number of grids at various distances (*p* < 0.001), as shown in the table (Table 1), consistent with the results of the viewing angle experiment. The experiment was replicated underwater, resulting in a significant decrease in the number of observed grids, indicating the influence of the different medium. Nevertheless, the digital endoscope continued to exhibit more grids at all distances (*p* < 0.001).

### 3.4. Standard for Mobile Imaging Architecture (SMIA) TV Distortion

**Figure 5:**
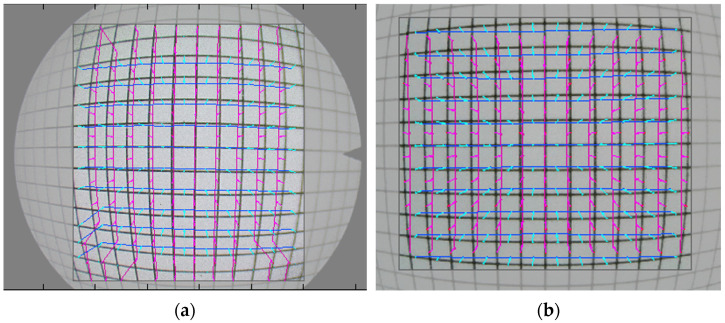
Distortion of the optic endoscope. The figure shows the distortions of the (**a**) optic and (**b**) digital endoscopes. The pink line represents the vertical deformation, while the light green line represents the horizontal deformation. The optic endoscope had slightly greater image distortion than the digital endoscope.

The calculation of SMIA TV distortion, started by Nokia and STMicroelectronics in 2004, is slightly different from that for a traditional distortion. The image captures for the grid test were also sent for examination for distortion. Usually, a larger visual field us usually accompanied by a larger distortion. Both images showed a barrel-type distortion. The ratio of distortion was −16.0 ± 0.87% for the digital endoscope and −17.6 ± 0.65% for the optic endoscope (Figure 5). This indicates lesser image distortion for the digital endoscope (*p* < 0.001), despite a larger visual field.

### 3.5. Modulation Transfer Function (MTF) and Spatial Frequency Response (SFR) Test

**Figure 6:**
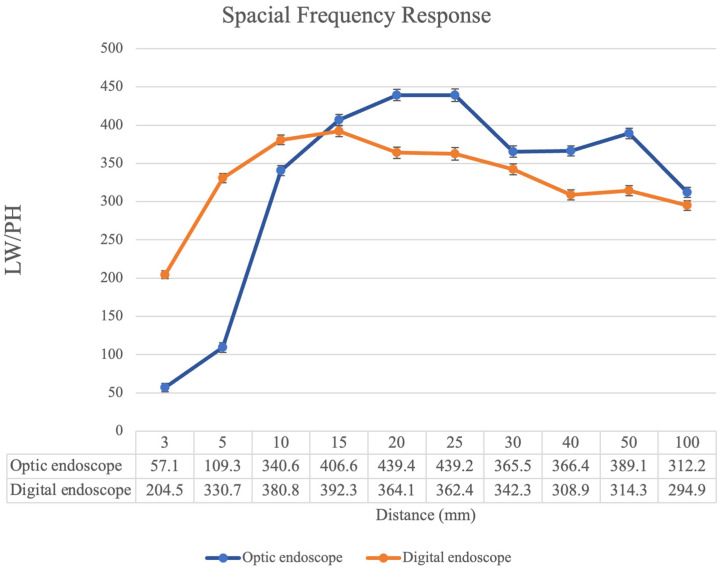
The spatial frequency response (SFR) graph illustrates the SFR, with the x-axis denoting distance (mm) and the y-axis representing LW/PH. The digital endoscope exhibited superior resolution within 10 mm, while the optic endoscope was preferred for longer distances.

MTF serves as a metric for assessing the capacity of an optical system to accurately replicate contrast across various spatial frequencies. It gauges the system’s ability to convey object details from the object space to the image space. Conversely, SFR, depicted graphically, is a specific representation of MTF. The SFR chart images were captured across distances ranging from 3 mm to 100 mm (Figure 6). The resolution of the endoscope at different distances can be measured by the ratio of line width to lines per height (LW/PH). The findings indicated superior performance of the digital endoscope within a 10 mm range, while the traditional endoscope was superior beyond a 15 mm distance.

### 3.6. 1951 United States Air Force (USAF) Resolution Test

The USAF test chart consists of a series of black and white line pairs arranged in groups. Each group represents a different level of spatial frequency. The resolution of the optical system is determined by the group and element numbers. The USAF chart typically provides information on the resolution in lp/mm for each group and element. The resolutions of the optic endoscope and digital endoscope are listed below (Table 2). The digital endoscope showed better or equal resolution within a 20 mm distance.

### 3.7. Color Difference

**Figure 7:**
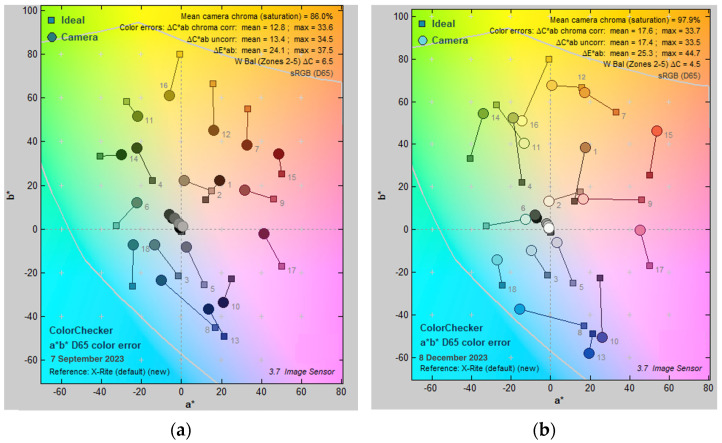
The color difference of the (**a**) optic endoscope and (**b**) digital endoscope. In the figure, the *x*-axis represents a* indicating color on a scale ranging from green to red. The *y*-axis represents b*, signifying color on a scale from blue to yellow. Each gray number on the chart represents a different color group, ranging from 1 to 18. Squares represent the original colors of the chart, while circles represent the colors displayed by the endoscope. The digital endoscope displayed a larger color difference. The overlapping circles in the middle of the diagram represent the color differences in grayscale, which cannot be clearly depicted in the image. Therefore, a separate discussion on ‘grayscale’ is presented in another paragraph in Section 3.9.

Color difference was assessed using a color checker, employing two values for the evaluation of color disparity. The value of Δ*C* gauges the difference in chromaticity between two colors, while Δ*E* provides a more comprehensive measure of overall color difference, considering variations in both chromaticity and luminance. The optic endoscope exhibited a Δ*C* of 13.1 ± 0.34, whereas the digital endoscope recorded 17.7 ± 0.42. In terms of Δ*E*, the optic endoscope registered 24.4 ± 0.54, while the digital endoscope showed 25.3 ± 0.50 (Figure 7). The color difference of the digital endoscope was greater than that of the optic endoscope (*p* < 0.001).

### 3.8. Luminance

**Figure 8:**
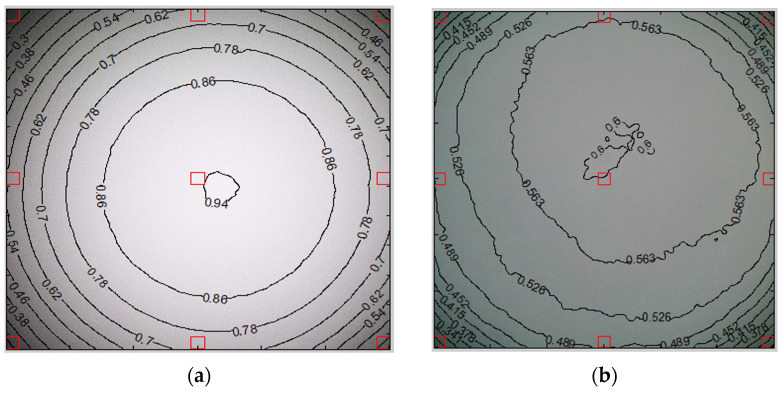
Luminance contour plot for (**a**) the optical endoscope and (**b**) the digital endoscope. The red rectangles represent the central region, as well as the four side and four corner regions. The black line represents identical luminance contour. The corners of the optical endoscope’s field of view are significantly darker than the center. Conversely, the luminance along the periphery of the digital endoscope is comparatively more uniform compared to the center.

Backlight images were captured for both endoscopes, and a luminance contour plot was generated (Figure 8). The luminance contour plot was generated by displaying normalized pixel level contours for the luminance channel of the image file. The plots depict the least and average corner values in unnormalized pixel levels and as a percentage of the maximum. The mean value was 50 ± 2.41% for the digital endoscope, in contrast to 29 ± 2.16% for the optical endoscope. This outcome indicates that the digital endoscope exhibits a more uniform luminance compared to the optical endoscope (*p* < 0.001).

### 3.9. Grayscale Analysis

**Figure 9:**
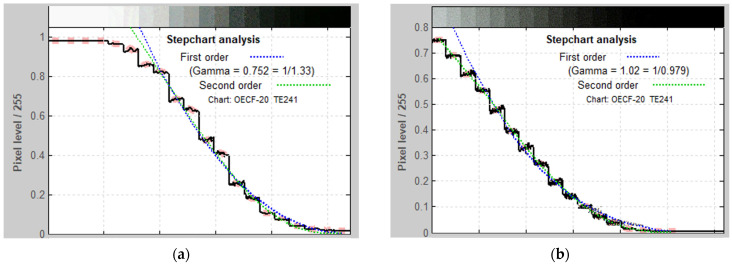
The images depict the outcomes of the grayscale stepchart analysis for both (**a**) the optic endoscope and (**b**) the digital endoscope. The *x*-axis represents the color scale from white to black, while the *y*-axis denotes the pixel level. The black curve represents the mean normalized pixel level of the patches, while the dashed blue and green curves illustrate the first and second order density fits, respectively. Gamma is derived from the first-order fit. Thick pale pink bars highlight the specific areas that were actively analyzed within the figure. The deficiencies of the optic endoscope are challenging to discern in most dark areas, as the pixel levels are the same for the last four scales.

Grayscale stepcharts were captured using both endoscopes, followed by analysis. The results revealed that the optic endoscope performed better in white areas but struggled in black areas. In contrast, the digital endoscope displayed a more balanced performance, showing consistent results in both areas. The optic endoscope successfully identified 17 different grayscales, whereas the digital endoscope accurately identified all 20 scales. The results showed that the digital endoscope demonstrated superior discernment of grayscale, as illustrated in Figure 9.

## 4. Discussion

Our research illustrates that the single-use digital spinal endoscope is a promising alternative to traditional optic endoscopes. Its disposable nature, convenience, broader visual field, and high image quality position the digital endoscope favorably for competition with traditional optic endoscopes (Table 3). To the best of our knowledge, there are few comparable designs currently available in the market, and none of them have had a thorough comparison with traditional endoscopes conducted. Our encouraging preliminary results may indicate an evolution in spine endoscopic surgery.

Surgical site infections pose a constant and severe risk for all surgeries, particularly in spine surgery. Infections around the spine and central nervous system can be challenging to treat and may result in serious consequences, including permanent deformities, neurological deficits, and even fatalities [34]. Consequently, infection prevention has become a paramount concern for spine surgeons. However, post-surgical infections are sometimes linked to incomplete disinfection of surgical tools. Traditional chemical disinfection methods for endoscopes, though long-standing, carry the potential risk of inadequate disinfection [35]. This concern has been highlighted in numerous previous studies, and cases of nosocomial infections following endoscopic procedures have been reported [36,37]. The intricate structure of endoscopes makes manual cleaning, especially within the working and irrigation channels, challenging [38]. The effectiveness of disinfection is also influenced by the duration of instrument soaking in the detergent and the thoroughness of manual cleaning, introducing human factors [39]. In the aftermath of the COVID-19 pandemic, the adoption of single-use medical devices has become mainstream to prevent cross-infections [40]. Considering its inherently intricate design and challenging cleaning process, endoscopes may be regarded as disposable devices [41]. A standardized sterilization process ensures the lowest pathogen load on the device, thus mitigating the risk of nosocomial surgical site infections [42]. The single-use duodenal endoscope has been proven to reduce the risk of patient infection [43], and the U.S. FDA also recommends the use of disposable duodenal endoscopes [44]. We believe that a similar effect can be observed in the case of spinal endoscopy.

Throughout the surgical procedure, there are numerous potential movements that can pose a threat to the delicate endoscope. Improper bending of the endoscope can result in deformation of its body. Accidental collisions between the endoscope and instruments or hard tissues may lead to lens scratches and cracks, thereby obscuring the surgeon’s vision. When electrical burrs or drills come too close to the lens, they can cause severe damage. Even with regular use, issues such as machine malfunctions, poor wire contact, lens fogging, or water leaking into the connection between the proximal lens and CCD can occur from time to time [45,46]. All these situations can disrupt surgeries, and some may not be quickly resolved. In such instances, surgeons may be compelled to alter the surgical procedure, potentially leading to legal issues. The single-use design of the endoscope addresses this by making it replaceable, thereby minimizing the risks associated with mechanical malfunctions. The water-proof design, devoid of additional connectors, also reduces the chances of water leaks and fogging. These features contribute to enhanced patient safety and facilitate the surgeon’s task. The manufacturing cost of the electronic endoscope prototype is approximately 100 USD, which is significantly cheaper compared to the typical optical endoscope with prices ranging from thousands to tens of thousands of dollars. Additionally, conventional optical endoscopes have a life cycle of around 50–100 surgeries [47]. Therefore, the electronic endoscope appears to be a cost-effective design.

The digital endoscope boasts a considerable advantage in terms of the visual field area and viewing angle, crucial factors for surgeons who anticipate a larger field of view during operations [14,48]. Accidental injury to structures outside the visual field can result in serious complications. Our digital endoscope not only exhibits a superior viewing angle in both horizontal and perpendicular directions but also outperforms the optical endoscope in the diagonal direction. The grid test with the endoscope in the air primarily simulates the situation where the endoscope enters the body without the infusion of a flushing solution. On the other hand, the condition in water simulates the scenario in typical surgeries where the field of view is filled with irrigation fluid. Both tests confirmed the advantage of the digital endoscope in terms of its field of view. The inherent square-shaped image produced by the digital endoscope aligns better with current monitor designs. In contrast, the image from the optical endoscope is circular, leaving a significant portion of the monitor in darkness. Typically, a larger field of view inevitably accompanies greater image torsion [49], and excessive image distortion may impact the precision of surgery, especially in spine surgeries that demand high accuracy. However, the distortion in the digital endoscope’s image is less than that of the optical endoscope. This allows the image to faithfully reflect the actual shape of structures, thereby enhancing the precision of the operation. The luminance at the peripheral region of the visual field is also superior in the digital endoscope. This more even distribution of luminance can be attributed to the endoscope’s design. LEDs are positioned on both sides of the endoscope tip, allowing for a more uniform illumination compared to traditional endoscopes, which typically have only one light source.

Both digital and optic endoscopes exhibit strengths in spatial resolution at varying distances. Due to the inherent design of digital endoscopes, their focal length may not extend as far as that of optic endoscopes. Consequently, when considering longer distances, the resolution of digital endoscopes may not match that of optic ones. However, in clinical practice, the typical working distance between the subject and the spine endoscope is usually small, while longer distances are rarely utilized in spine endoscope surgery. It is difficult to determine superiority or inferiority between the two types of endoscopes within this range. The superior resolution of the digital endoscope at close distances assists in distinguishing tissues during surgery, minimizing the potential for unintentional harm to nerves or vessels. Additionally, it benefits surgeons in identifying pathological lesions from normal areas. An additional advantage of the digital endoscope is its automatic focus capability, eliminating the need for manual adjustments based on distance required by optic endoscopes. This not only saves surgeons trouble but also allows them to free up their hands for other tasks.

The color difference is more pronounced in the digital endoscope, and we attribute this difference to our intentional adjustments. In surgical procedures, bleeding is a frequent occurrence, and the image may be intermittently obscured by red [14,50]. Even with minor oozing, the overall color of the image tends to shift toward the red spectrum. To alleviate visual fatigue for surgeons and enable them to concentrate on active bleeding, we have intentionally adjusted the intensity of the red-light signal. We believe this adjustment is responsible for the observed increase in color difference. It is important to note that this modification does not compromise the image quality and can be personalized based on individual preferences.

The existing digital endoscope serves as a prototype and has yet to be implemented in actual surgical procedures, whether in animal studies or real-world scenarios. Prior to its introduction into clinical practice, it is imperative to conduct a comprehensive evaluation encompassing safety and effectiveness assessments. These evaluations will encompass the efficacy of sterilization processes, the biocompatibility of materials used, and rigorous durability tests. To reduce the potential for unwanted patient reactions, we prioritized the use of biocompatible materials that meet ISO 10993 requirements for the endoscope [51]. The endoscope underwent rigorous processes to ensure its sterility and biocompatibility before being utilized on patients, in compliance with ISO 11135 and 11737, which address sterilization and bacterial endotoxin control for medical devices [52,53]. Additionally, the disposable digital endoscope complies with essential electrical safety standards and undergoes systematic software lifecycle processes, as required by IEC 60601 and IEC 62304 [54,55]. Subsequent stages involve the orchestration of animal experiments, cadaveric studies, and clinical trials, ensuring strict compliance with Institutional Animal Care and Use Committee (IACUC) and Institutional Review Board (IRB) regulations. We plan to conduct in vitro experiments on animal tissues to simulate the in vivo environment. Additionally, we will verify safety in live experimental animals and simulate actual spine endoscopic surgery in cadaveric experiments. Beyond these foundational steps, numerous applied technologies, such as exoscope, 3D imaging, virtual reality (VR), augmented reality (AR), and navigation-guided surgery hold the potential to enhance endoscopic techniques and streamline surgical procedures [56]. Our future trajectory aims to integrate these cutting-edge technologies, aiming to elevate the overall performance of our digital endoscope. We also intend to improve the resolution at longer distances and enhance color presentation to meet clinical requirements. While enhancing the quality of healthcare, we should also consider the impact on the environment. Choosing environmentally friendly and recyclable materials, reducing carbon emissions during the manufacturing process, and limiting disposables to the portions directly in contact with patients are all methods to reduce environmental impact [57]. This comprehensive approach not only contributes to an improved patient care experience but also helps create a more sustainable healthcare system.

There are still limitations to our current study. This spine endoscope prototype is in the early stages and requires additional scrutiny and refinement for eventual commercialization. Before its application in actual surgeries, further in vitro and animal experiments are essential. We solely utilized a single optic endoscope as a reference, and this may not accurately reflect the features of endoscopes from different manufacturers. Conducting additional comparative studies with various commercial endoscopes is essential. Additionally, selecting the right material for the product is crucial to strike a balance between patient benefits and environmental protection. Despite these considerations, we believe that the design of this single-use digital endoscope is a trend and may address the shortcomings of traditional optic endoscopes.

## 5. Conclusions

The present study demonstrates that a single-use spine endoscope can deliver an image quality comparable to, or even surpassing, that of traditional optic endoscopes. The substantial advantages of a larger field of view and disposability make it a promising option for spine surgery. Further exploration is warranted to implement this design in actual spine surgical procedures.

## Figures and Tables

**Table 1:** Grid ground test results in different mediums. The table shows the number of grids observed at different distances. The number under the digital endoscope heading is the grid number in the horizontal and vertical directions.

Medium	Distance	Optic Endoscope (Mean ± SD)	Digital Endoscope (Mean ± SD)	*p* Value
Air	10 mm	6.8 ± 0.13	8.2 ± 0.14	<0.001
Air	20 mm	13.2 ± 0.16	16.0 ± 0.19	<0.001
Air	30 mm	20.1 ± 0.19	23.8 ± 0.21	<0.001
Water	10 mm	4.3 ± 0.10	5.2 ± 0.10	<0.001
Water	20 mm	8.2 ± 0.12	9.5 ± 0.14	<0.001
Water	30 mm	11.3 ± 0.15	14.0 ± 0.13	<0.001

**Table 2:** Calculated resolution for the optic endoscope: The first number represents the minimum group number that can be identified. The second number represents the number of line pairs per millimeter in the USAF Resolving Power Test. The number in brackets indicates the corresponding resolution.

Distance	Optic Endoscope	Digital Endoscope
3 mm	3–4 (11.30 lp/mm)	4–4 (22.62 lp/mm)
5 mm	3–6 (14.3 lp/mm)	4–2 (17.95 lp/mm)
10 mm	3–5 (12.7 lp/mm)	3–5 (12.7 lp/mm)
15 mm	3–2 (8.98 lp/mm)	3–3 (10.10 lp/mm)
20 mm	3–1 (8 lp/mm)	3–1 (8 lp/mm)

**Table 3:** Overview of the contrast between digital and optical endoscopes.

	Digital Endoscope	Optic Endoscope
Weight	Lighter (39 g)	Heavier (214 g)
Endoscope characteristics	Single-use	Repetitive use
Field of view	Larger (100–131°)	Smaller (89°)
Shape of visual field	Square	Circle
Distortion	Smaller (−16.0%)	Larger (−17.6%)
Resolution	Better at close distances	Better at longer distances
Color difference	Larger (Δ*C*: 17.7, Δ*E*: 25.3)	Smaller (Δ*C*: 13.1, Δ*E*: 24.4)
Grey scale	20 levels	17 levels
Luminance	Even	Central

## Data Availability

The data used to support the finding of this study are available from the corresponding author upon request. The data are not publicly available due to confidentiality agreements.
